# Mycobacterium Lysine ε-aminotransferase is a novel alarmone metabolism related persister gene via dysregulating the intracellular amino acid level

**DOI:** 10.1038/srep19695

**Published:** 2016-01-25

**Authors:** Xiangke Duan, Yunsong Li, Qinglin Du, Qinqin Huang, Siyao Guo, Mengmeng Xu, Yanping Lin, Zhidong Liu, Jianping Xie

**Affiliations:** 1 Institute of Modern Biopharmaceuticals, State Key Laboratory Breeding Base of Eco-Environment and Bio-Resource of the Three Gorges Area, key laboratory of Eco-environment three gorges reservoir, Ministry of Education, School of Life Sciences, Southwest University. Chongqing 400715, China; 2 Hanhong College, Southwest University. Chongqing 400715, China; 3 Department of thoracic surgery, Beijing Tuberculosis and Thoracic Tumor Research Institute, Beijing Chest Hospital, Capital Medical University, Beijing 101149, China

## Abstract

Bacterial persisters, usually slow-growing, non-replicating cells highly tolerant to antibiotics, play a crucial role contributing to the recalcitrance of chronic infections and treatment failure. Understanding the molecular mechanism of persister cells formation and maintenance would obviously inspire the discovery of new antibiotics. The significant upregulation of *Mycobacterium tuberculosis* Rv3290c, a highly conserved mycobacterial lysine ε-aminotransferase (LAT) during hypoxia persistent model, suggested a role of LAT in persistence. To test this, a *lat* deleted *Mycobacterium smegmatis* was constructed. The expression of transcriptional regulator leucine-responsive regulatory protein (LrpA) and the amino acids abundance in *M. smegmatis lat* deletion mutants were lowered. Thus, the persistence capacity of the deletion mutant was impaired upon norfloxacin exposure under nutrient starvation. In summary, our study firstly reported the involvement of mycobacterium LAT in persister formation, and possibly through altering the intracellular amino acid metabolism balance.

Bacterial persistence, a ubiquitous bacterial physiological state phenotypically tolerant to antibiotics, is increasingly recognized as the culprit of the intractability of chronic and relapsing infections. Bacterial persisters are specialized survivors genetically identical to nontolerant kins but under a non-growing or extremely slow-growing, non-replicating dormant state^1^. Sequestration of the targets instead of targets mutation contribute to the antibiotics tolerance of persister^2^. Persister cells are stochastically developed during mid-exponential phase^3^ under favorable conditions^4^. Despite more than 70 years after the first report of persisters^5^, the molecular mechanisms underlying bacterial persistence remain largely elusive. Several pathogens, such as *Mycobacteria*, *Pseudomonas* and *Staphylococcus*^6^,^7^,^8^, are remarkable persisters.

*Mycobacteria* have two notorious pathogens, *M. tuberculosis* (*Mtb*) for tuberculosis and *M. leprae* for leprosy, and nontuberculous *Mycobacterium* (NTM)^9^. Many *M. tuberculosis* genes have been reported to be associated with persister formation and essential for persistence in mouse model, such as those involved in energy metabolism^10^,^11^,^12^,^13^,^14^, *RelA* (ppGpp synthase)^15^, *M. tuberculosis PhoY2*, a homolog of *E.coli PhoU*^16^,^17^, RpsA (S1 protein), a protein involved intrans-translation, proteasome PrcBA^18^,^19^, Toxin-antitoxin (TA) modules^20^,^21^,TA locus *vapBC*^22^, *RelE*^20^, other 88 putative TA system candidates in *M. tuberculosis* genome and 30 TA modules^23^,^24^,^25^,^26^,^27^. The differential expression of *Rv3290c* in persisters^27^,^28^ implicates an important role in persistence.

Lysine ε-aminotransferase (LAT) is a pyridoxal 5′-phosphate(PLP)-dependent enzyme that converts l-lysine to α- aminoadipate-δ-semialdehyde and glutamate, which is subsequently converted to A^l^-piperideine-6-carboxylic acid^29^,^30^. In the β-lactam-producing *Actinomycetes*, LAT has been shown to catalyze the first steps of β-lactam antibiotic biosynthesis pathway^31^. *Rv3290c*, encoding a Lysine ε-aminotransferase (LAT) in *M. tuberculosis*, was upregulated over 40-fold in nutrient-starved persistence models^32^. In this study, we initially reported the involvement of LAT in mycobacterium persister formation.

## Materials and Methods

### Antibiotics

Ampicillin, kanamycin, hygromycin, norfloxacin were bought from Sangon Biotech Co., and their stock solutions were freshly prepared, filter-sterilized, and used at indicated concentrations.

### Bacterial culture and starvation conditions

The bacterial strains and plasmid used in this study are shown in Table 1. *E.coli* strains were grown on LB broth agar or in LB broth, *Mycobacterium smegmatis* mc^2^155 was grown in 7H9 liquid medium (Difco) supplemented with 0.05% w/v Tween 80, 0.5%glycerol and 0.5%glucose or were grown on 7H10 agar supplemented with 1% glycerol and 0.5% glucose. The starvation culture condition as described^32^,^33^ with minor modifications. In brief, exponential phase cultures were pelleted and washed twice with 1 × PBS before being resuspended in 1 × PBS, transferred to standing flasks or microwell and incubated at 37 °C, 110 rpm. For viability determination during starvation, bacteria were cultured in 50 ml volumes in 250 ml bottles (Shuniu), and the number of cfu/ml was determined by plating serial dilutions onto 7H10 agar from triplicate cultures at several time points (0 h, 24 h, and 72 h).

When required, the following antibiotics were used at the final concentration: ampicillin,100 μg/ml; kanamycin, 500 μg/ml for *E.coli* or 200 μg/ml for *M. smegmatis*; hygromycin, 50 μg/ml.

### Knockout mutant construction and complementation

The *lat* gene of *M. smegmatis*mc^2^155 was disrupted using specialized transduction previously described^34^. PCR and sequencing of *lat*_*Msm*_ was used to confirm the deletion. For knockout mutant complementation, the *M. tuberculosis* H37Rv *Rv3290c* coding region was amplified by polymerase chain reaction using the primers: 5′-CGCGGATCCGCGTCCTGCTATCATAGCGTCATG-3′, bearing a *Bam*H I restriction site followed by the start codon of *Rv3290c*; and 5′-GGAATTCCATATGGAATTCCGGCTGCCTTACGTCACCAC-3′, consisting of the final three C-terminal amino acids of *Rv3290c* and a TAA termination codon followed by a *Nde* I restriction site. The gel-purified polymerase chain reaction product was digested with *Bam*H I and *Nde* I, yielding a 1395-base pair *Bam*H I/*Nde* I fragment. The fragments were ligated into the plasmid pALACE digested by *Bam*H I and *Nde*I, to produce the plasmid pALACE-*lat*_*Mtb*_. Sequencing of pALACE-*lat*_*Mtb*_ confirmed the correctness of the constructed fragment. Competent cells of *lat*_*Msm*_ mutant were prepared as described^34^, and pALACE containing *lat*_*Mtb*_ gene was used to transform *lat*_*Msm*_ mutant competent cells. This was followed by electroporation^35^ into the mutant as previously described^34^. Transformed cells were streaked on 7H10 plates containing 100 μg/ml ampicillin and 50 μg/ml hygromycin. The desired complemented strain was identified by bacterial PCR and Western Blotting. To detect His-tagged LAT_Mtb_, bacterial pellets were harvested and disrupted by ultrasonication. Samples were then subjected to SDS-PAGE and the His-tagged LAT_Mtb_ protein was detected by mouse anti-His antibodies (TIANGEN, China).

### Amino acids determination

Overnight cultures of *M. smegmatis* mc^2^155 were diluted 1:100 in M9 medium and incubated at 37 °C on shaker (PEIYING DHZ-CA, TAI CANG SHI YAN SHE BEICHANG) at 110 rpm. Exponential phase cultures were harvested by centrifugation at 8000 rpm, 4 °C for 15 min. Harvested cells were washed three times with ddH_2_O and resuspended in 5 ml of ddH_2_O. Then the bacteria were pipetted into the dialysis tube and dialyzed in ddH_2_O at 4 °C for 24 h. Cells were collected and transferred to freeze-dried reagent bottle for freeze dehydration by vacuum pump. The procedure was from reference^36^ with slight modification. Briefly, 50 mg dried sample was put into a 15 * 150 mm testtube, and then 6 ml of 6 M HCl were added into the testtube containing bacterial cells. The upper part in the testtube was removed and the testtube was sealed after 10 min vacuumization. The treated testtube was hydrolyzed for 22 hours in a 110 °C ± 1 °C oven. The testtube was taken out and cooled to room temperature, mixed and filtered.1 ml of filtrate was put into a 50 ml beaker, and waterbathing evaporated at 60 °C, 2-fold diluted by adding 0.02 M HCl, the sample was filtered by 0.22 um membrane, and loaded into a Hitachi L-8800 amino acid analyzer. The analysis cycle is 53 min, using two columns during the analysis process: (1) Separation column: (4.6 mm × 60 mm) Eluent flow rate is 0.4 ml/min, the column temperature was 70 °C, column pressure was 11.627 MPa. (2) Reaction column: Ninhydrin and ninhydrin buffer flow was 0.35 ml/min, the column temperature was 135 °C, column pressure was 1.078 MPa.

### MIC assay and drug treatment of cultures

The MICs of antibiotics were determined by using serial two-fold dilution of the antibiotics in 7H9 medium as previously described in reference^37^. The initial cell densities were 10^8^cfu/ml of exponential culture, and the samples were incubated for 3 days at 37 °C.The MIC was recorded as the minimum drug concentration abolishing visible growth. For drug treatment assay, 50 ml *M. smegmatis* was cultured under starvation conditions in 250 ml bottles. At 0, 24 h and 72 h time point, one milliliter of a starvation culture of *M. smegmatis* was diluted 10 times in 1 × PBS, and 1 ml was aliquoted per well of a 48-well plate, every sample with 3 repeats. Norfloxacin was added to duplicate wells of cultures at final concentration of 20 μg/ml. Control wells for cultures received no drug or no cells. Cultures were incubated with or without drug at 37 °C for 24 h and 48 h, followed by serial diluting and plating on 7H10 agar to determine bacterial viability.

### RNA Isolation and reverse transcription-PCR (RT-PCR)

*M. smegmatis* cultured under starvation conditions in 50 ml volumes in 250 ml bottles (Shuniu).Three 50 ml cultures were harvested by centrifugation after 24 h and 72 h of starvation. Control samples were prepared by washing log-phase cultures twice with PBS then resuspended in PBS, as described for the preparation of starved bacteria, and harvesting 50mlculture by centrifugation at time zero (t = 0). Pellets were pulverized in liquid nitrogen and homogenized in Trizol solution (Invitrogen) and RNA was isolated according to the manufacturer’s instructions. The subsequent steps were performed according to the reference^38^.

### Real-Time PCR

1 μg of total RNA was reversely transcribed to cDNA using a first strand cDNA synthesis kit (Roche) according to the manufacturer’s instructions. Resultant cDNA was used for real-time PCR. Advanced SYBRGreen Supermix (BIO-RAD) were used to quantify cDNA in a 20 μL reaction containing 10 μM each primer, 10 μl supermix (2X), 4 μL cDNA. Primers used are listed in Table 1. Copy numbers of *MSMEG_1764* mRNA were normalized with copy numbers of *sigA* mRNA. Each reaction was run in triplicate in Bio-Rad CFX-96 Real-Time Detection System with the following parameters: 95 °C for 2 Min, 40 cycles of 95 °C for 10 Sec, and 64 °C for 40 Sec.

## Results

### *lat* is conserved among *Mycobacteria*

**Figure 1:**
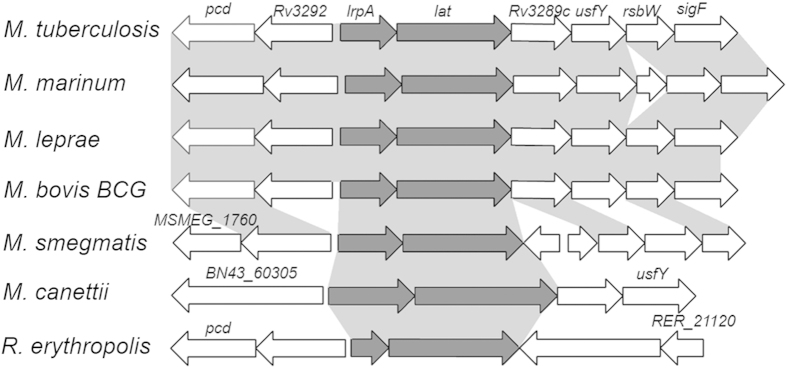
Genomic context of *lat* among *Mycobacteria* and close relatives. Arrows represented with a dark grey background correspond to genes conserved among all species, whereas genes without ortholog in at least one species are shown with white arrows.

*lat* is conserved among *M. tuberculosis*, *M. marinum*, *M. leprae*, *M. bovis* BCG, *M. smegmatis*, *M. canettii*, and *Rhodococcus erythropolis PR4* by BLAST analysis. The amino acid identity between *M. tuberculosis* LAT and its homologs is greater than 59% in all cases, its neighboring gene *lrpA* is conserved too. Most neighboring genes of *M. tuberculosis*, *M. marinum*, *M. leprae*, and *M. bovis* BCG are highly conserved (Fig. 1).

### *lat*_*Msm*_ is upregulated under nutrient starvation in *M. smegmatis*

**Figure 2:**
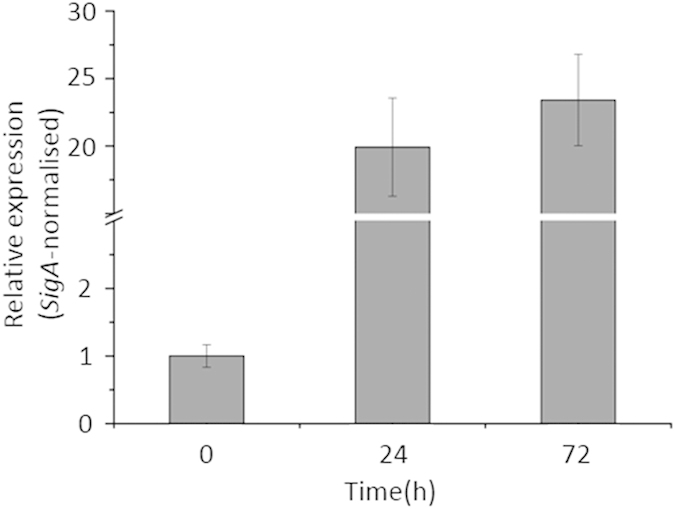
The expression of *M. smegmatis lat* under nutrient starvation. Cells of *M. smegmatis* were initially grown in 7H9 medium to log-phase and then washed by 1 × PBS and resuspended in 1 × PBS, incubate at 37 °C, 110 rpm. RNAs were extracted from bacteria harvested at indicated time points. RT-PCR was performed as described in Materials and Methods. Data are means ± s.d. of triplicates in one of at least three experiments.

*MSMEG_1764* is the homolog of *Rv3290c* in *M. smegmatis* and shares 77% identity with *Rv3290c*. Since *lat* was highly upregulated in *M. tuberculosis* under nutrient starvation, it is interesting to know whether this is the case in *M. smegmatis*. To this end, a nutrient starvation model according to Betts *et al.*^39^ was established. The transcription of *lat* in *M. smegmatis* at 0 h, 24 h and 72 h was measured. *lat* was upregulated 20 and 23-fold in *M. smegmatis* undergoing starvation after 24 and 72 hours, respectively (Fig. 2). The results showed that the expression pattern of *M. smegmatis lat* under nutrient starvation is the same as that in *M. tuberculosis*. Given *M. smegmatis* is a well-recognized facile surrogate to address *M. tuberculosis* biology^40^, in particular to study the persistence under nutrient starvation^41^, *M. smegmatis* was adopted as a model in our study.

### *lat* (*MSMEG_1764*) is nonessential and can be deleted in *M. smegmatis* mc^2^155

**Figure 3:**
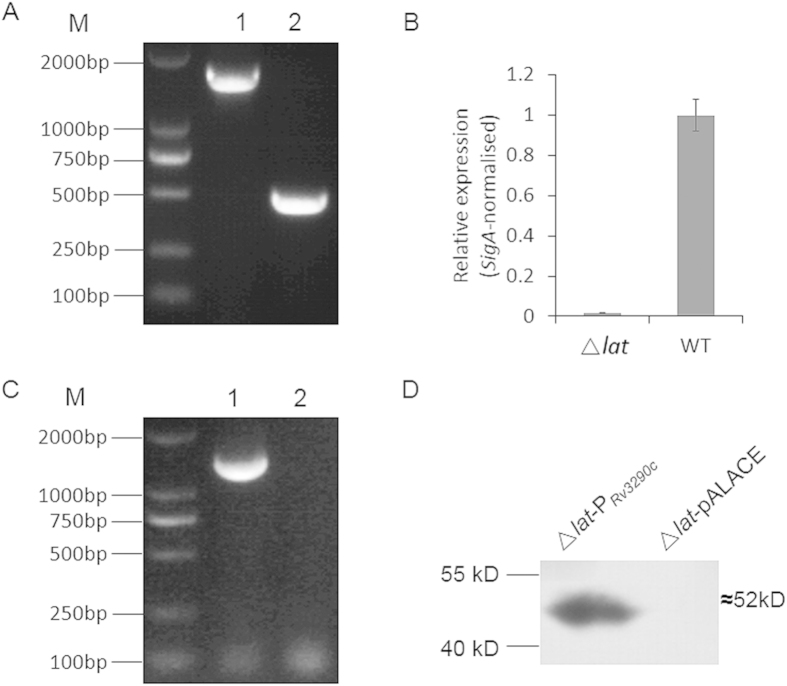
Construction of *MSMEG_1764* knockout mutant and complement strains. (**A**) PCR verification of the construction *MSMEG_1764* knockout strain. Lanes: 1.Wild-type MS; 2. *MSMEG_1764* knockout strain. (**B**) Verify the transcription of *MSMEG_1764* knockout strain by RT-PCR. Wild type and Δ*lat*_*Msm*_ strains were grown at 37C in MB 7H9 liquid medium to an OD600 of 0.8–1.0. Total bacterial RNA was isolated and subjected to RT-PCR to detect the expression of the *lat*_*Msm*_ gene. (**C**) Construction of Δ*lat-Rv3290c* strain; Lanes: 1. Knockout strain complement with pALACE-*Rv3290*c; 2. Knockout strain complement with pALACE plasmid. (**D**) Western-blotting to confirm the expression of Rv3290c.Lysates were prepared from bacterial cells cultured as in (**B**), after 16 h induction and subjected to Western blotting to detect His-tagged Rv3290c protein using mouse anti-His antibody.

To determine the effect of *lat* mutation on the persistence of mycobacterium, a *lat* knockout mutant was constructed by *Xer* site-specific recombination as described in the Materials and Methods section. *M. smegmatis* hygromycin-resistant colonies were selected and cultured consecutively for five generations. Primer lat-F1 and lat-R1 were then used to confirm the mutant genotype, with the wild-type strain as a control. The 1565 bp (1365 bp of the wild type *lat*_*MS*_ and extra 200 bp from the genome) fragment can be amplified from the wild-type, while there was only 449 bp (249 bp of Δ*lat*_*MS*_ and extra 200 bp from the genome) amplicon from the *lat*_*Msm*_ knockout mutant (Fig. 3A). The *lat*_*Msm*_ knockout mutant was further confirmed by the absence of transcription product (Fig. 3B), indicating that the Δ*lat* mutant of *M. smegmatis* was successfully constructed. Blastp shows that *MSMEG_1764* is the homolog of *Rv3290c* in *M. smegmatis* and shares 77% identity with *Rv3290c*. Hence, we use *M. tuberculosis Rv3290c* gene for complementation assays. The recombination vector pALACE-*Rv3290c* in which *Rv3290c* is under the control of an ACE promoter was transformed into the Δ*lat* mutant strain to get the complementary strain. Bacteria PCR result shows that a 1350 bp band was amplified in the complementary strain and no band for the parental strain (Fig. 3C).Western Blotting analysis using the anti-His antibody further confirmed the presence of the expressed ~52 kDa LAT_Mtb_-His fusion protein in the cell lysates of the complemented strain, while absent in the parental strain (Fig. 3D).

### *lat* deleted *M. smegmatis*is is hypersensitive to norfloxacin and shows declined persistence under nutrient starvation

**Figure 4:**
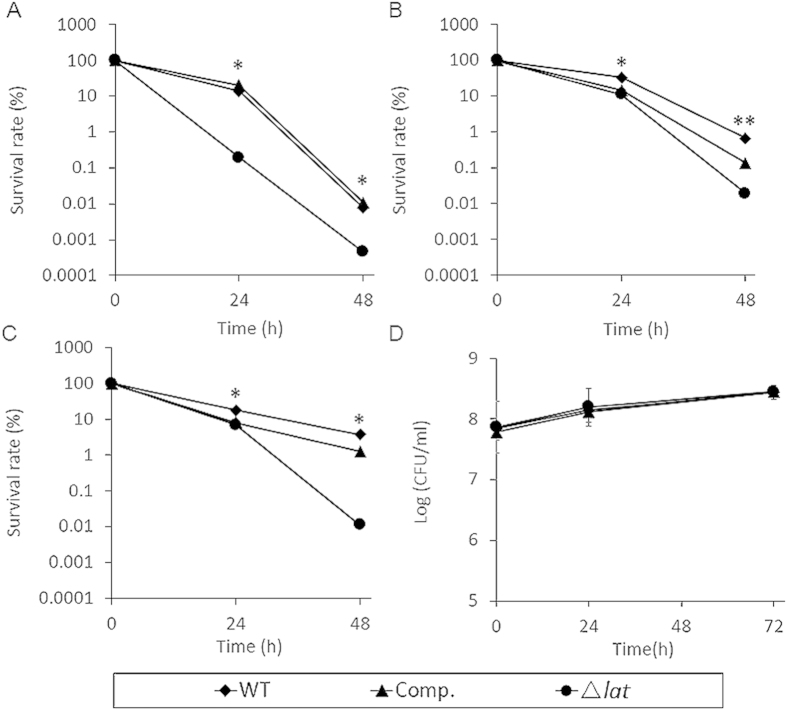
Survival of strains under norfloxacin exposure in 1 × PBS buffer. Wild type, complement strain and Δ*lat*_*Msm*_ strain treated with norfloxacin after 0 h (**A**), 24 h (**B**) and 72 h (**C**) starvation. Data are shown as means ± SD of triplicate wells. Similar results were obtained in three independent experiments. (**D**) Starvation time and persister formation. Log phase cultures of wild type, complement and *lat*_*Msm*_ knockout strain were washed by 1 × PBS and then cultured under starvation condition. Data points are averages of 3 independent experiments. **P < 0.01, *P < 0.05, Student’s *t* test, significant difference from the Δ*lat*_*MS*_ and the wild type strain.

LAT was profoundly upregulated during *M. tuberculosis* starvation persistence model. The effect of *lat* deletion on *M. smegmatis* persistence remains elusive. To test this, we first measured the MIC of norfloxacin, rifampicin and isoniazid, the MIC value showed that the deletion of *lat* has no effect upon the antibiotic sensitivity for the *M. smegmatis* wild-type and complement strain (2 μg·ml^−1^ for norfloxacin, 8 μg·ml^−1^ for rifampicin and 4 μg·ml^−1^ for isoniazid). The deletion of genes involved in persistence usually has no effect upon MIC^42^,^43^. To test the effect of *lat* deletion on the MIC, the strains were exposed to high concentration antibiotics. The survival rate of Δ*lat*_*Msm*_ strain is lower than wild-type and complement strain under 20 μg·ml^−1^ norfloxacin exposure (Fig. 4A).

**Figure 5:**
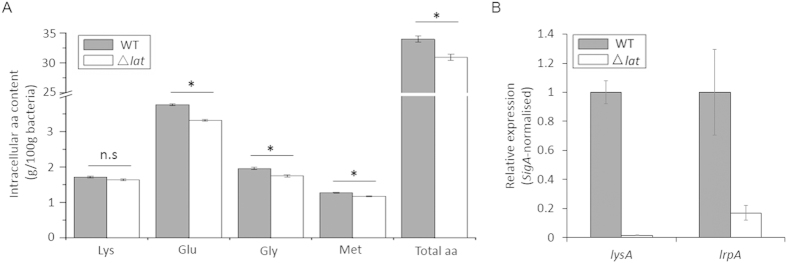
Deletion of *lat*_*Msm*_ resulted in the decrease of intracellular amino acid content. (**A**) Determination of intracellular amino acid content in WT and Δ*lat*_*Msm*_ strain. (**B**) The transcription level of *lysA* and *lrpA* in wild type and Δ*lat*_*Msm*_ strains. Data are shown as means ± SD of triplicate wells. Similar results were obtained in three independent experiments. n.s indicates no significant difference (P ≥ 0.1), *P < 0.05, Student’s *t* test, significant difference from the Δ*lat*_*MS*_ and the wild type strain.

Since LAT was significantly upregulated under nutrient starvation in *M. tuberculosis*. It is interesting to know whether the deletion of *lat* will compromise the survival of *M. smegmatis* under starvation. Therefore, we tested the viability of mutant strain in 1 × PBS buffer as described by Betts *et al.*^39^. As shown in Fig. 5, no difference can be seen between wild-type and Δ*lat*_*Msm*_ strain after 72 hours starvation. To explore whether the deletion of *lat*_*Msm*_ only affect the persistence of mutant strain in high antibiotic concentrations, strains were subjected to antibiotic exposure and the ratio of persisters was determined. The 24 hours and 72 hours-starved cultures were exposed to norfloxacin for 48 h and the number of survived strains was assessed. We can see that 24 h and 72 h (Fig. 4B,C) starvation cultures of three strains exhibit higher level of norfloxacin persistence than 0 h time point. With the increase of starvation, wild type and complement strains persisted, but not Δ*lat*_*Msm*_ strain (Fig. 4D).Complementation of the mutant with *Rv3290c* restored the phenotype of wild type strain. After 24 h and 72 h starvation, all strains showed a higher tolerance to norfloxacin than non-starvation cultures. This result indicated that deletion of *lat*_*Msm*_ failed to affect the viability under nutrient starvation, but can impair the persistence of the mutant strain under norfloxacin treatment.

### Inactivation of *lat*_*Msm*_ decreased the intracellular amino acids content

^13^C-isotope profiling of the persisters of *Staphylococcus aureus* revealed an active amino acid anabolism in this subpopulation, including Ala, Asp, Glu, Ser, Gly and His^44^. Dysregulation of intracellular amino acids level has been shown to lower the survival capability within macrophage exemplified by *M. tuberculosis pkn*G mutants, which encodes protein serine/threonine kinase involved in the regulation of amino acids level^45^. To examine whether the inactivation of *MSMEG_1764* has an effect on the amount of intracellular amino acids, the intracellular levels of the amino acids were determined for the wild-type, mutant, and complemented strains. As shown in Fig. 5A, the concentration of glutamic acid, glycine, methionine and total amino acids content in the Δ*MSMEG_1764* mutant strain was decreased, but without significant differences for the lysine content between the mutant strain and the wild-type strains. This might be due to the feedback repression of lysA (Diaminopimelate (DAP) - decarboxylase, the enzyme involved in the last step of lysine biosynthesis) by the excess of lysine. This shows that the rate of lysine biosynthesis was regulated by the intracellular lysine amount^46^. To test whether *lysA* control the lysine level in *M. smegmatis*, we measured the expression level of *lysA* in these strains. As expected, the transcription of *lysA* in Δ*lat*_*Msm*_ strain was decreased (Fig. 5B). Genomic context analysis showed that there is a transcriptional regulator leucine-responsive regulatory protein (LrpA) upstream of *lat* (Fig. 2), a regulator capable of directly binding to the upstream region of *lat*^47^. To test whether the transcription of *lrpA* was also decreased, RT-PCR was applied. The result showed that *lrpA* transcription was also lowered in Δ*lat* strain (Fig. 5B).The results suggested that the deletion of *lat* in *M. smegmatis* decreased the intracellular amino acids amount and downregulated the transcription of *lrpA*.

### *relA*_*Msm*_ was downregulated in *lat*_*Msm*_ deletion strain under nutrient starvation

**Figure 6:**
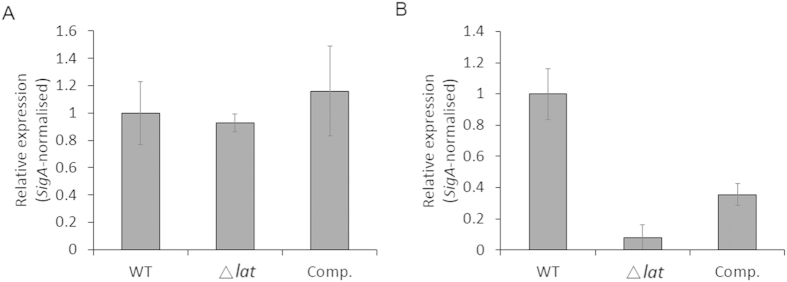
Response of the *rel*_*Msm*_ to starvation. Profile of the relative expression levels of *rel*_*Msm*_ across 0 h and 24 h time course. Data are means ± s.d. of triplicates in one of at least three experiments.

The stress alarmones guanosine 3′,5′-bispyrophosphate ((p)ppGpp), a key molecule in antibiotic resistance^48^ and bacterial persistence^49^, was found to accumulate rapidly under starvation. The relationship between *relA* and *lrpA* has previously been noted^50^,^51^.The expression of *lrp* is positively controlled by ppGpp, namely the lower ppGpp level means fewer Lrp^50^. Moreover, the expression of *lrp* was significantly reduced in the strains failed to produce ppGpp^50^. Rel_Mtb_, the *M. tuberculosis* bifunctional enzyme responsible for both (p)ppGpp synthesis and hydrolysis^52^, and has been shown to play an important role in the survival of bacteria during nutrient starvation condition^53^. To test whether the downregulation of *lrpA* resulted from the decreased expression of *Rel* in *M. smegmatis*, the transcription level of *rel*_*Msm*_, the *rel*_*Mtb*_ homolog of *M. smegmatis*^54^, was measured at different intervals under starvation. No significant difference can be spotted between wild type and knockout strain at 0 h starvation (Fig. 6A). But *rel*_*Msm*_ was markedly downregulated in Δ*lat*_*Msm*_ strain after 24 h starvation (Fig. 6B).Complementation of *M. smegmatis* Δ*lat*_*MSm*_ with the *M. tuberculosis lat Rv3290c* can partially restore the expression of *rel*_*Msm*_. This result suggested that knockout *lat*_*Msm*_ has no effect upon the intracellular (p)ppGpp content under normal conditions, but can influence the synthesis and hydrolysis of (p)ppGpp under nutrient starvation.

## Discussion

Bactericidal antibiotics usually can sterilize most bacteria rapidly. However, a sub-population will survive and be reactivated upon the withdrawal of the antibiotics^55^. Genes involving in the persisters formation are intensively studied^56^ both for fundamental insights and translational medicine ends.

Here we showed that *lat* gene *MSMEG_1764*, the homologue of Mtb *lat Rv3290c* is involved in the persister formation (specifically tolerance to norfloxacin) via mediating the intracellular amino acid contents and altering the expression of ppGpp synthase expression in *M. smegmatis*.

Inactivation of the homologue of *Rv3290c* in *M. smegmatis* leads to more sensitive to norfloxacin than the wild type strain (Fig. 4), but without change to the MIC (Data not shown). Generally, mutation of genes involved in persister formation should not alter the MIC, but only affect the persistence^42^,^43^. LAT was previously showed to be involved in the L-lysine metabolism^57^. The data here showed that the knockout of *lat*_*Msm*_ rendered a decrease of intracellular total amino acid, but without discernable effect on the accumulation of L-lysine (Fig. 5A). LAT is just one of the several genes involved in *M. tuberculosis* glutamate metabolism^58^, given the diversity and redundancy of genes to control the robustness of this important amino acid homeostasis, it is not quite unexpected that the disruption of LAT only slightly decreased the glutamate content in *M. smegmatis*.

Leucine-responsive regulatory protein (Lrp) is a global transcriptional regulator widespread among prokaryotes, and modulates the expression of a variety of genes involved in metabolism during starvation, especially in the amino acid catabolism and anabolism^59^,^60^. The knockout of *lrp* in *E.coli* downregulated the expression of the amino acid metabolism-related genes^61^. In our study, we found the expression of *lrpA* was downregulated in *lat*_*Msm*_ knockout strain (Fig. 5B), which can explain why the intracellular amino acid content was decreased in knockout mutants. Previous study showed that *lrpA* is profoundly up-regulated during nutrient starvation conditions characteristic of persistent/latent phase in *M. tuberculosis*^39^, as well as in *M. smegmatis* (Fig. 5B). The persistence of *lrpA* inactivated *Mycobacterium fortuitum* was attenuated in a murine infection model^62^. The expression of *lrp* is stimulated by (p)ppGpp and LRP can regulate the biosynthesis of amino acid^50^. Here we have shown that the deletion of *lat*_*Msm*_ downregulated the expression of *rel*_*Msm*_ (Fig. 6). *M. tuberculosis* failed to produce (p)ppGpp under starvation was defective in long-term survival both *in vitro*^53^ and *in vivo*^15^. In our study, the downregulation of *rel*_*Msm*_ is coincident with the diminished bacterial tolerance to antibiotics.

**Figure 7:**
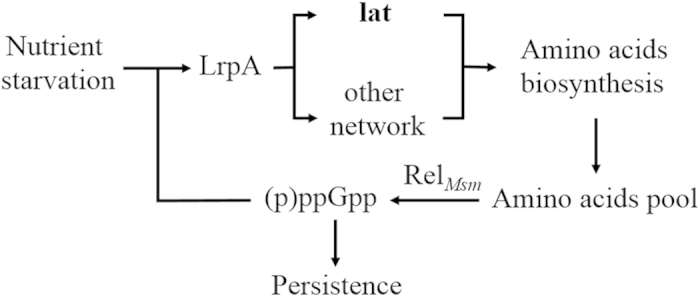
A model of feed-forward loop controlling *M. smegmatis* persistence in response to nutrition starvation .

In conclusion, we have shown that LAT is involved in persister formation in mycobacteria. Under nutrition starvation, (p)ppGpp is produced by Rel_*Msm*_ and accumulates within bacteria, then upregulates the transcription of *Lrp*A gene, which in turn upregulates the protein level of LAT and response for amino acid metabolism. Intracellular amino acid level regulates the accumulation of (p)ppGpp, and (p)ppGpp controls the dormant persister formation (Fig. 7). The *lat* deleted mutant strain failed to replenish the amino acid pool, then downregulated the synthesis of (p)ppGpp. This is largely due to the positive regulation of the transcription of *lrpA* by (p)ppGpp. The determination of the *M. tuberculosis* H37Rv LAT crystal structure and the identification of its active sites^63^ will promisingly facilitate the discovery of novel LAT inhibitors^64^. Our finding has shown that LAT is a new player in mycobacterial persistence provided a potential drug target for inhibitors against persisters.

## Additional Information

**How to cite this article**: Duan, X. *et al.* Mycobacterium Lysine ε-aminotransferase is a novel alarmone metabolism related persister gene via dysregulating the intracellular amino acid level. *Sci. Rep.* **6**, 19695; doi: 10.1038/srep19695 (2016).

## Figures and Tables

**Table 1:** Bacterial strains, plasmids, and primers used in this study.

Description	Genotype or relevant phenotype	Source or reference
Strains		
*E.Coli* DH5α	supE44 Δlac*U169*(φ80lacZΔM15)*hsdR17recA1 endA1 gyrA96thi-1 relA1*λpir	
*M. smegmatis* mc^2^155	High-frequency transformation mutant of *M. smegmatis* ATCC 607	
Plasmids		
PET-28	Kanamycin resistance plasmid	
pALACE	*E. coli–Mycobacteria* shuttle vector, hygromycin resistance plasmid	38
PJV53	Expression of Che9c gp61, Kanamycin resistance plasmid	34
PAL-75	Providing hygromycin resistance cassette	34
Primers		
LAT-F	5′-CGCGGATCCGCGTCCTGCTATCATAGCGTCATG-3′	This study
LAT-R	5′-GGAATTCCATATGGAATTCCGGCTGCCTTACGTCACCAC-3′	
KO-F1	5′-GCACTAGTGGGGTTTGTCGGTGGTGAT-3′	This study
KO-R1	5′-CGAAGATCTCCAGGAAACGCTAGATCTTCCGCGCGTCGACCAGATGCG-3′	
KO-F2	5′-ACAAGATCTAGCGTTTCCTGGAGATCTGTCCGGCGCTGCCCGTGA-3′	This study
KO-R2	5′-GCAGAAGCTTATCACATCTCCCGGCTCG-3′	
lat-F1	5′-TGCTTCATCGCCGAACCCATCCA-3′	This study
lat-R1	5′-CGAACGAGACCACATCGGGCATCACA-3′	
lrpA-F	5′-ATTCTGGTCCGCGAACTCGTGGCA-3′	This study
lrpA-R	5′-CGACGAACGCCGAAAGCATGTTGC-3′	
rel-F	5′-AAGTCATTGCCCCACTTGCCCATC-3′	This study
rel-R	5′-CGGCCCTTGACGATCATCTTCTGGT-3′	
lysA-F	5′- CAGAAGTTCGGTTTGTCGCTGGCCA -3′	This study
lysA-R	5′-GCTTGTCGGCCAGTTCCTTCATG -3′	
sigA-F	5′-AAGACACCGACCTGGAACTC-3′	38
sigA-R	5′-AGCTTCTTCTTCCTCGTCCTC-3′	

## References

[b1] Lewis K. Persister Cells. Annual Review of Microbiology 64, 357–372 (2010). 10.1146/annurev.micro.112408.134306 20528688

[b2] Keren I., Kaldalu N., Spoering A., Wang Y. & Lewis K. Persister cells and tolerance to antimicrobials. FEMS Microbiol Lett 230, 13–18 (2004). 14734160 10.1016/S0378-1097(03)00856-5

[b3] Balaban N. Q., Merrin J., Chait R., Kowalik L. & Leibler S. Bacterial persistence as a phenotypic switch. Science 305, 1622–1625 (2004). 15308767 10.1126/science.1099390

[b4] Dorr T., Lewis K. & Vulic M. SOS response induces persistence to fluoroquinolones in Escherichia coli. PLoS Genet 5, e1000760 (2009). 20011100 10.1371/journal.pgen.1000760 PMC2780357

[b5] Bigger J. Treatment of staphylococcal infections with penicillin by intermittent sterilisation. The Lancet 244, 497–500 (1944).

[b6] Gengenbacher M. & Kaufmann S. H. Mycobacterium tuberculosis: success through dormancy. FEMS Microbiology Reviews 36, 514–532 (2012). 22320122 10.1111/j.1574-6976.2012.00331.x PMC3319523

[b7] Harrison J. J., Turner R. J. & Ceri H. Persister cells, the biofilm matrix and tolerance to metal cations in biofilm and planktonic Pseudomonas aeruginosa. Environmental Microbiology 7, 981–994 (2005). 15946294 10.1111/j.1462-2920.2005.00777.x

[b8] Chong Y. P. *et al.* Persistent Staphylococcus aureus bacteremia: a prospective analysis of risk factors, outcomes, and microbiologic and genotypic characteristics of isolates. Medicine 92, 98–108 (2013). 23429353 10.1097/MD.0b013e318289ff1e PMC4553980

[b9] Cohen N. R., Lobritz M. A. & Collins J. J. Microbial persistence and the road to drug resistance. Cell Host Microbe 13, 632–642 (2013). 23768488 10.1016/j.chom.2013.05.009 PMC3695397

[b10] Bryk R. *et al.* Selective killing of nonreplicating mycobacteria. Cell Host Microbe 3, 137–145 (2008). 18329613 10.1016/j.chom.2008.02.003 PMC2423947

[b11] Dhiman R. K. *et al.* Menaquinone synthesis is critical for maintaining mycobacterial viability during exponential growth and recovery from non‐replicating persistence. Molecular microbiology 72, 85–97 (2009). 19220750 10.1111/j.1365-2958.2009.06625.x PMC4747042

[b12] Dhar N. & McKinney J. D. Mycobacterium tuberculosis persistence mutants identified by screening in isoniazid-treated mice. Proc Natl Acad Sci U S A 107, 12275–12280 (2010). 20566858 10.1073/pnas.1003219107 PMC2901468

[b13] Deb C. *et al.* A novel in vitro multiple-stress dormancy model for Mycobacterium tuberculosis generates a lipid-loaded, drug-tolerant, dormant pathogen. PLoS One 4, e6077 (2009). 19562030 10.1371/journal.pone.0006077 PMC2698117

[b14] McKinney J. D. *et al.* Persistence of Mycobacterium tuberculosis in macrophages and mice requires the glyoxylate shunt enzyme isocitrate lyase. Nature 406, 735–738 (2000). 10963599 10.1038/35021074

[b15] Dahl J. L. *et al.* The role of RelMtb-mediated adaptation to stationary phase in long-term persistence of Mycobacterium tuberculosis in mice. Proc Natl Acad Sci U S A 100, 10026–10031 (2003). 12897239 10.1073/pnas.1631248100 PMC187750

[b16] Li Y. & Zhang Y. PhoU is a persistence switch involved in persister formation and tolerance to multiple antibiotics and stresses in Escherichia coli. Antimicrob Agents Chemother 51, 2092–2099 (2007). 17420206 10.1128/AAC.00052-07 PMC1891003

[b17] Shi W. & Zhang Y. PhoY2 but not PhoY1 is the PhoU homologue involved in persisters in Mycobacterium tuberculosis. J Antimicrob Chemoth 65, 1237–1242 (2010). 10.1093/jac/dkq103 PMC2868530 20360062

[b18] Shi W. *et al.* Pyrazinamide inhibits trans-translation in Mycobacterium tuberculosis. Science 333, 1630–1632 (2011). 21835980 10.1126/science.1208813 PMC3502614

[b19] Gandotra S., Schnappinger D., Monteleone M., Hillen W. & Ehrt S. In vivo gene silencing identifies the Mycobacterium tuberculosis proteasome as essential for the bacteria to persist in mice. Nat Med 13, 1515–1520 (2007). 18059281 10.1038/nm1683 PMC3174471

[b20] Singh R., Barry C. E. 3rd & Boshoff H. I. The three RelE homologs of Mycobacterium tuberculosis have individual, drug-specific effects on bacterial antibiotic tolerance. J Bacteriol 192, 1279–1291 (2010). 20061486 10.1128/JB.01285-09 PMC2820853

[b21] Black D. S., Irwin B. & Moyed H. S. Autoregulation of hip, an operon that affects lethality due to inhibition of peptidoglycan or DNA synthesis. J Bacteriol 176, 4081–4091 (1994). 8021189 10.1128/jb.176.13.4081-4091.1994 PMC205607

[b22] Demidenok O. I., Kaprelyants A. S. & Goncharenko A. V. Toxin-antitoxin vapBC locus participates in formation of the dormant state in Mycobacterium smegmatis. FEMS Microbiol Lett 352, 69–77 (2014). 24417293 10.1111/1574-6968.12380

[b23] Rosenberg S. M., Ramage H. R., Connolly L. E. & Cox J. S. Comprehensive Functional Analysis of Mycobacterium tuberculosis Toxin-Antitoxin Systems: Implications for Pathogenesis, Stress Responses, and Evolution. PLoS Genetics 5, e1000767 (2009). 20011113 10.1371/journal.pgen.1000767 PMC2781298

[b24] Hu Y. *et al.* Detection of mRNA transcripts and active transcription in persistent Mycobacterium tuberculosis induced by exposure to rifampin or pyrazinamide. J Bacteriol 182, 6358–6365 (2000). 11053379 10.1128/jb.182.22.6358-6365.2000 PMC94781

[b25] Muttucumaru D. G., Roberts G., Hinds J., Stabler R. A. & Parish T. Gene expression profile of Mycobacterium tuberculosis in a non-replicating state. Tuberculosis (Edinb) 84, 239–246 (2004). 15207493 10.1016/j.tube.2003.12.006

[b26] Kesavan A. K., Brooks M., Tufariello J., Chan J. & Manabe Y. C. Tuberculosis genes expressed during persistence and reactivation in the resistant rabbit model. Tuberculosis (Edinb) 89, 17–21 (2009). 18948063 10.1016/j.tube.2008.08.004 PMC2655131

[b27] Keren I., Minami S., Rubin E. & Lewis K. Characterization and Transcriptome Analysis of Mycobacterium tuberculosis Persisters. mBio 2, e00100-00111-e00100-00111 (2011). 10.1128/mBio.00100-11 PMC3119538 21673191

[b28] Voskuil M. I., Visconti K. C. & Schoolnik G. K. Mycobacterium tuberculosis gene expression during adaptation to stationary phase and low-oxygen dormancy. Tuberculosis (Edinb) 84, 218–227 (2004). 15207491 10.1016/j.tube.2004.02.003

[b29] Soda K., Misono H. & Yamamoto T. L-Lysine - Alpha-Ketoglutarate Aminotransferase .I. Identification of a Product Delta1-Piperideine-6-Carboxylic Acid. Biochemistry 7, 4102-& (1968). 5722275 10.1021/bi00851a045

[b30] Soda K. & Misono H. L-Lysin - Alpha-Ketoglutarate Aminotransferase .2. Purification Crystallization and Properties. Biochemistry 7, 4110-& (1968). 5722276 10.1021/bi00851a046

[b31] Fujii T., Narita T., Agematu H., Agata N. & Isshiki K. Characterization of L-lysine 6-aminotransferase and its structural gene from Flavobacterium lutescens IFO3084. J Biochem-Tokyo 128, 391–397 (2000). 10965037 10.1093/oxfordjournals.jbchem.a022766

[b32] Betts J. C., Lukey P. T., Robb L. C., McAdam R. A. & Duncan K. Evaluation of a nutrient starvation model of Mycobacterium tuberculosis persistence. Mol Microbiol 43, 717–731 (2002). 11929527 10.1046/j.1365-2958.2002.02779.x

[b33] Albrethsen J. *et al.* Proteomic profiling of Mycobacterium tuberculosis identifies nutrient-starvation-responsive toxin-antitoxin systems. Mol Cell Proteomics 12, 1180–1191 (2013). 23345537 10.1074/mcp.M112.018846 PMC3650330

[b34] van Kessel J. C. & Hatfull G. F. Mycobacterial recombineering. Methods Mol Biol 435, 203–215 (2008). 18370078 10.1007/978-1-59745-232-8_15

[b35] Parish T. & Stoker N. G. Electroporation of mycobacteria. In: Mycobacteria protocols (ed^(eds). Springer (1998).

[b36] Novikova A. E. S. N., Sycheva E. V. & Kolokolova A. V. Identification and analysis of norvaline and norleucine in fermentation broths of E. coli strains. Sorption Chromatogr Proc 6, 796–806 (2006).

[b37] Ribeiro A. L. *et al.* Analogous mechanisms of resistance to benzothiazinones and dinitrobenzamides in Mycobacterium smegmatis. PLoS One 6, e26675 (2011). 22069462 10.1371/journal.pone.0026675 PMC3206020

[b38] Du Q., Long Q., Mao J., Fu T., Duan X. & Xie J. Characterization of a novel mutation in the overlap of tlyA and ppnK involved in capreomycin resistance in Mycobacterium. IUBMB Life 66, 405–414 (2014). 24890219 10.1002/iub.1277

[b39] Betts J. C., Lukey P. T., Robb L. C., McAdam R. A. & Duncan K. Evaluation of a nutrient starvation model of Mycobacterium tuberculosis persistence by gene and protein expression profiling. Mol Microbiol 43, 717–731 (2002). 11929527 10.1046/j.1365-2958.2002.02779.x

[b40] Jacobs W. R., Tuckman M. & Bloom B. R. Introduction of foreign DNA into mycobacteria using a shuttle phasmid. Nature 327, 532–535 (1987). 3473289 10.1038/327532a0

[b41] Smeulders M. J., Keer J., Speight R. A. & Williams H. D. Adaptation of Mycobacterium smegmatis to stationary phase. J Bacteriol 181, 270–283 (1999). 9864340 10.1128/jb.181.1.270-283.1999 PMC103559

[b42] Hansen S., Lewis K. & Vulic M. Role of global regulators and nucleotide metabolism in antibiotic tolerance in Escherichia coli. Antimicrob Agents Chemother 52, 2718–2726 (2008). 18519731 10.1128/AAC.00144-08 PMC2493092

[b43] Manuel J., Zhanel G. G. & de Kievit T. Cadaverine suppresses persistence to carboxypenicillins in Pseudomonas aeruginosa PAO1. Antimicrob Agents Chemother 54, 5173–5179 (2010). 20855735 10.1128/AAC.01751-09 PMC2981224

[b44] Lechner S. *et al.* Metabolic and transcriptional activities of Staphylococcus aureus challenged with high-doses of daptomycin. Int J Med Microbiol 304, 931–940 (2014). 24980509 10.1016/j.ijmm.2014.05.008

[b45] Cowley S. *et al.* The Mycobacterium tuberculosis protein serine/threonine kinase PknG is linked to cellular glutamate/glutamine levels and is important for growth in vivo. Mol Microbiol 52, 1691–1702 (2004). 15186418 10.1111/j.1365-2958.2004.04085.x

[b46] Boy E., Richaud C. & Patte J. C. Multiple regulation of DAP‐decarboxylase synthesis in Escherichia coli K‐12. FEMS Microbiology Letters 5, 287–290 (1979).

[b47] Reddy M. C., Gokulan K., Jacobs W. R. Jr., Ioerger T. R. & Sacchettini J. C. Crystal structure of Mycobacterium tuberculosis LrpA, a leucine-responsive global regulator associated with starvation response. Protein Sci 17, 159–170 (2008). 18042675 10.1110/ps.073192208 PMC2144582

[b48] Wu J., Long Q. & Xie J. (p)ppGpp and drug resistance. J Cell Physiol 224, 300–304 (2010). 20432457 10.1002/jcp.22158

[b49] Maisonneuve E., Castro-Camargo M. & Gerdes K. (p)ppGpp controls bacterial persistence by stochastic induction of toxin-antitoxin activity. Cell 154, 1140–1150 (2013). 23993101 10.1016/j.cell.2013.07.048

[b50] Landgraf J. R., Wu J. & Calvo J. M. Effects of nutrition and growth rate on Lrp levels in Escherichia coli. J Bacteriol 178, 6930–6936 (1996). 8955316 10.1128/jb.178.23.6930-6936.1996 PMC178595

[b51] Tani T. H., Khodursky A., Blumenthal R. M., Brown P. O. & Matthews R. G. Adaptation to famine: A family of stationary-phase genes revealed by microarray analysis. Proceedings of the National Academy of Sciences of the United States of America 99, 13471–13476 (2002). 12374860 10.1073/pnas.212510999 PMC129697

[b52] Avarbock D., Salem J., Li L. S., Wang Z. M. & Rubin H. Cloning and characterization of a bifunctional RelA/SpoT homologue from Mycobacterium tuberculosis. Gene 233, 261–269 (1999). 10375643 10.1016/s0378-1119(99)00114-6

[b53] Primm T. P., Andersen S. J., Mizrahi V., Avarbock D., Rubin H. & Barry C. E. 3rd. The stringent response of Mycobacterium tuberculosis is required for long-term survival. J Bacteriol 182, 4889–4898 (2000). 10940033 10.1128/jb.182.17.4889-4898.2000 PMC111369

[b54] Jain V., Saleem-Batcha R., China A. & Chatterji D. Molecular dissection of the mycobacterial stringent response protein Rel. Protein Sci 15, 1449–1464 (2006). 16731979 10.1110/ps.062117006 PMC2242531

[b55] Spoering A. L. & Lewis K. Biofilms and planktonic cells of Pseudomonas aeruginosa have similar resistance to killing by antimicrobials. J Bacteriol 183, 6746–6751 (2001). 11698361 10.1128/JB.183.23.6746-6751.2001 PMC95513

[b56] Fauvart M., De Groote V. N. & Michiels J. Role of persister cells in chronic infections: clinical relevance and perspectives on anti-persister therapies. J Med Microbiol 60, 699–709 (2011). 21459912 10.1099/jmm.0.030932-0

[b57] Tripathi S. M. & Ramachandran R. Overexpression, purification and crystallization of lysine epsilon-aminotransferase (Rv3290c) from Mycobacterium tuberculosis H37Rv. Acta Crystallogr Sect F Struct Biol Cryst Commun 62, 572–575 (2006). 10.1107/S1744309106016824 PMC2243093 16754985

[b58] Cole S. T. *et al.* Deciphering the biology of Mycobacterium tuberculosis from the complete genome sequence. Nature 393, 537–544 (1998). 9634230 10.1038/31159

[b59] Calvo J. M. & Matthews R. G. The leucine-responsive regulatory protein, a global regulator of metabolism in Escherichia coli. Microbiol Rev 58, 466–490 (1994). 7968922 10.1128/mr.58.3.466-490.1994 PMC372976

[b60] Brinkman A. B., Ettema T. J., de Vos W. M. & van der Oost J. The Lrp family of transcriptional regulators. Mol Microbiol 48, 287–294 (2003). 12675791 10.1046/j.1365-2958.2003.03442.x

[b61] Traxler M. F. *et al.* Discretely calibrated regulatory loops controlled by ppGpp partition gene induction across the ‘feast to famine’ gradient in Escherichia coli. Mol Microbiol 79, 830–845 (2011). 21299642 10.1111/j.1365-2958.2010.07498.x PMC3073637

[b62] Parti R. P. S. *et al.* A transposon insertion mutant of Mycobacterium fortuitum attenuated in virulence and persistence in a murine infection model that is complemented by Rv3291c of Mycobacterium tuberculosis. Microb Pathogenesis 45, 370–376 (2008). 10.1016/j.micpath.2008.08.008 18930129

[b63] Mani Tripathi S. & Ramachandran R. Direct evidence for a glutamate switch necessary for substrate recognition: crystal structures of lysine epsilon-aminotransferase (Rv3290c) from Mycobacterium tuberculosis H37Rv. J Mol Biol 362, 877–886 (2006). 16950391 10.1016/j.jmb.2006.08.019

[b64] Dube D., Tripathi S. M. & Ramachandran R. Identification of in vitro inhibitors of Mycobacterium tuberculosis Lysine ε-aminotransferase by pharmacophore mapping and three-dimensional flexible searches. Medicinal Chemistry Research 17, 182–188 (2008).

